# miR-182-5p contributes to radioresistance in naso-pharyngeal carcinoma by regulating BNIP3 expression

**DOI:** 10.3892/mmr.2021.11935

**Published:** 2021-02-23

**Authors:** Wei He, Hongyan Jin, Qian Liu, Quanxin Sun

Mol Med Rep 23: 130, 2021; DOI: 10.3892/mmr.2020.11769

The authors have realized that, on p. 9 in the right-hand column, the second sentence featured in the final summary paragraph of the Discussion should have been deleted from the proofs prior to the publication of this article (the sentence that read as: ‘This result indicated that downregulated BNIP3 expression may decrease the radioresistance of NPC cells.’). Therefore, this sentence has been eliminated from the paper. The authors are grateful to the editor of *Molecular Medicine Reports* for allowing them to publish this Corrigendum, and all the named authors agree to its contents. The authors also apologize to the readership for any inconvenience caused.

